# The Expansion of House Mouse Major Urinary Protein Genes Likely Did Not Facilitate Commensalism with Humans

**DOI:** 10.3390/genes14112090

**Published:** 2023-11-17

**Authors:** Miloš Macholán, Kristina Daniszová, Zuzana Hiadlovská

**Affiliations:** 1 Institute of Animal Physiology and Genetics, Laboratory of Mammalian Evolutionary Genetics, Czech Academy of Sciences, 602 00 Brno, Czech Republic; 2 Department of Botany and Zoology, Faculty of Science, Masaryk University, 601 77 Brno, Czech Republic

**Keywords:** copy number variation, ddPCR, MUP excretion, *Mus musculus*, proteomics, synanthropy

## Abstract

Mouse wild-derived strains (WDSs) combine the advantages of classical laboratory stocks and wild animals, and thus appear to be promising tools for diverse biomedical and evolutionary studies. We employed 18 WDSs representing three non-synanthropic species (*Mus spretus*, *Mus spicilegus*, and *M. macedonicus*) and three house mouse subspecies (*Mus musculus musculus*, *M. m. domesticus*, *M. m. castaneus*), which are all important human commensals to explore whether the number of major urinary protein (MUP) genes and their final protein levels in urine are correlated with the level of commensalism. Contrary to expectations, the MUP copy number (CN) and protein excretion in the strains derived from *M. m. castaneus*, which is supposed to be the strongest commensal, were not significantly different from the non-commensal species. Regardless of an overall tendency for higher MUP amounts in taxa with a higher CN, there was no significant correlation at the strain level. Our study thus suggests that expansion of the *Mup* cluster, which appeared before the house mouse diversification, is unlikely to facilitate commensalism with humans in three house mouse subspecies. Finally, we found considerable variation among con(sub)specific WDSs, warning against generalisations of results based on a few strains.

## 1. Introduction

Advancements in life sciences are inevitably contingent on biological models. Besides well-known and extensively employed invertebrates such as the nematode *Caenorhabditis elegans* or *Drosophila* fruit flies, the house mouse (*Mus musculus*) is undoubtedly one of the most widespread model organisms. This synanthropic mammal species consists of three widespread subspecies: *musculus*, *domesticus*, and *castaneus*, which diverged approximately 350–500 thousand years ago [1,2,3,4,5]. House mice are significant human commensals, while other species involved in the same clade (‘*M. musculus* group’: *M. spretus*, *M. macedonicus*, *M. spicilegus*, and *M. cypriacus*) live for the most part outside the synanthropic niche [6,7].

Like most other models, mice have several advantages. They are relatively easy to breed and manipulate, have a relatively short generation time, and can reproduce all year round. In fact, the entire genome sequence of the house mouse was published only a year after that of humans [8], and large databases of well-annotated sequences and diverse molecular markers with known positions in the genome are now available. Since house mice are highly tolerant of inbreeding, numerous inbred laboratory stocks have been established during the last century. However, these ‘classical laboratory strains’ (CLSs) have several substantial limitations.

First, these mice represent an artificial taxon. In the 1980s, it became apparent that most CLSs, until then considered domesticated *M. m. domesticus*, carry *M. m. musculus* Y chromosomes [9,10,11]. Later, it was shown that CLSs’ genome is a mixture of all three subspecies, though the contribution of *domesticus* prevails [12,13,14]. Second, the genetic and phenotypic diversity of CLSs is severely restricted. For example, despite a wide array of existing stocks, their ancestry basically traces back to a single female [12,15,16,17]. Therefore, mouse inbred strains cannot encompass the whole variation in the wild and do not represent natural conditions.

Consequently, scientists have started to pay attention to wild mice and more natural settings in addressing genetic, physiological, behavioural, and ecological questions, e.g., using seminatural enclosures [18,19,20,21,22,23] or barns [24,25,26]. However, the scale of natural variation covered by the mice in these experiments has necessarily been limited. Moreover, high variability contradicts the requirements for experimental reproducibility (see [13] for a review of the strengths and limitations of CLSs and wild mice).

A promising compromise between the availability of sufficient variation and reproducibility is inbred stocks directly derived from mice captured in natural populations [17,27,28,29]. Contrary to CLSs, the geographic origin and pedigree of these wild-derived strains (WDSs) are precisely known. Although they cannot equal wild mice in the level of variation that they harbour, this deficiency can be alleviated by increasing the number of strains employed. A current study of 101 WDSs representing five species (including the three *M. musculus* subspecies) and eight natural Y chromosome consomic strains has revealed substantially higher variation than CLSs; for example, WDSs displayed as many as 2483% single-nucleotide polymorphisms (SNPs) compared to CLSs [17]. Therefore, WDSs appear to be excellent tools for diverse genetic, behavioural, and ecological studies, allowing miscellaneous hypothesis testing.

Ideal candidates for such testing are genes involved in olfaction. The house mouse is extraordinary because it possesses ~1200 olfactory receptors and ~250 pheromone receptors in its genome [30,31,32]. The high numbers of receptors mirror a complex mix of chemical signals involved in mouse communication. This mix includes products of multigene families such as secretoglobins (also known as androgen binding proteins, ABPs), the major histocompatibility complex (MHC), exocrine gland-secreted proteins (ESPs), odorant-binding proteins (OBPs), and major urinary proteins (MUPs) (see [33,34,35] for review), detected either by the main olfactory epithelium or vomeronasal organ.

Our study focuses specifically on major urinary proteins, which play a prominent role in mouse chemical communication [36]. These relatively small (18–20 kDa) barrel-shaped lipocalin proteins bind low-molecular-mass ligands, including volatile pheromones, which are released through urine, saliva, tears, and other secretions into the external environment. Because of this bond, the release is slow, and the effect of the ligands is thus protracted [37,38]. However, at least some of them can, on their own, also modulate the recipients’ behaviour and physiology [39,40,41,42,43,44]. The most important are urinary MUPs (uMUPs) which are expressed in the liver and secreted through the kidneys into urine [45,46,47]. The uMUP production is sexually dimorphic: males excrete 3–10 times more uMUPs than females [43,48,49].

In house mice, the *Mup* cluster consists of at least 21 genes and 20 pseudogenes, which are tandemly arrayed across an ~2 Mb long stretch of chromosome 4. There are two classes of *Mup* genes that differ in sequence similarity and expression profiles. Evolutionarily older is the peripheral class, characterised by <82% mature protein sequence identity. The central cluster is younger and highly homogeneous (>97% mature protein sequence identity), suggesting a very recent series of duplications or gene conversions [50,51,52]. According to Sheehan et al. (2019), the central *Mup* cluster has undergone two phases of expansion: a minor one in the common ancestor of *M. musculus*, *M. spretus*, *M. macedonicus*, and *M. spicilegus,* and a second, larger expansion predating the diversification of the three *M. musculus* subspecies.

The timing of the second central *Mup* expansion suggests that this event may have facilitated the evolution of human commensalism in house mice about 10,000 years ago [52]. This is because the ability to convey complex information through urinary scent marks can be beneficial in densely populated synanthropic environments [53]. High population densities associated with the synanthropic niche should increase interaction rates among mice, making information-rich urinary marks highly important. Therefore, although the expansion of the central *Mup* gene cluster predated human commensalism, its further diversification could have been positively selected by the synanthropic bond [52]. On the other hand, we may assume that *Mup* genes, like other large gene families, are prone to high within-species copy number variation (CNV) [54,55]. Unfortunately, a large part of this variation remains undetected despite years of high-throughput sequencing. One of the reasons for this gap is difficulties with correctly assembling large repeated regions [56,57], whereas other approaches like qPCR may lack adequate accuracy [58]. Moreover, most previous studies have been limited to a couple of CLSs, such as the reference genome mouse C57BL/6, while information on the number and arrangement of *Mup* paralogs in wild populations and/or other mouse species is rather scarce. This gene cluster may thus harbour an important portion of undetected variation [50,51].

As mentioned above, the number of *Mup* genes is positively correlated with the level of synanthropy in the *M. musculus* group, with a higher CN in the commensal house mouse than in other, non-commensal species [52]. This raises the question of whether this association can be extended to different house mouse subspecies or populations. For example, according to Payne et al. (2001) [59] and Beynon et al. (2002) [60], a free-living (‘feral’) island *M. m. domesticus* population revealed higher MUP profile similarity and lower diversity than the mainland, farm-living populations. Similarly, Cheetham et al. (2009) [49] reported reduced MUP profile complexity and diversity in lab strains compared to wild mice. While the former case is likely to be largely related to population size differences, and all three studies were focused on MUP electrophoretic band profiles rather than the number of *Mup* genes, these findings suggest that variations in total gene count and expression can be expected not only between synanthropic and non-synanthropic mouse species, but also among subspecies and populations of *M. musculus* itself.

Despite the fact that house mice exhibit high ecological plasticity [61,62], *M. m. domesticus* is generally believed to be more tightly associated with humans than *M. m. musculus* [61,63,64,65]. Furthermore, populations of the former subspecies were shown to be more strongly structured into local breeding units or demes [66]. Much less information is available regarding the Asian subspecies *M. m. castaneus*, but it is also known to be highly commensal [61,67,68,69] or even more commensal than *M. m. domesticus* [61,63,65], being “man‘s closest indoor associate among undomesticated mammals” [70] (p. 20).

In this study, we test for a potential association between the level of synanthropy and the number of *Mup* gene copies within the *M. musculus* species group using the droplet digital PCR method. These data are complemented with measurements of MUP levels in urine. Wild individuals are not suitable for this purpose because each mouse is genetically distinct (and, for some part, heterozygous), which hampers generalisations. Therefore, here, we employ 18 WDSs representing three house mouse subspecies and three other species of *Mus*. We show that notwithstanding a general trend for a higher CN in *M. musculus* than in its non-commensal relatives, CNV does not fully reflect the strength of synanthropy. Moreover, though the total numbers of the urinary *Mup* gene copies were found to be correlated with their overall uMUP levels in the urinary proteome, CN is a poor predictor of the final level of uMUP excretion in individual strains. Finally, we show that variation among strains derived from different subspecies does not exceed variation within subspecies. We thus confirm the high genetic variability harboured by WDSs and hence their great value for use in many fields of life sciences.

## 2. Materials and Methods

### 2.1. Mice

**Figure 1:**
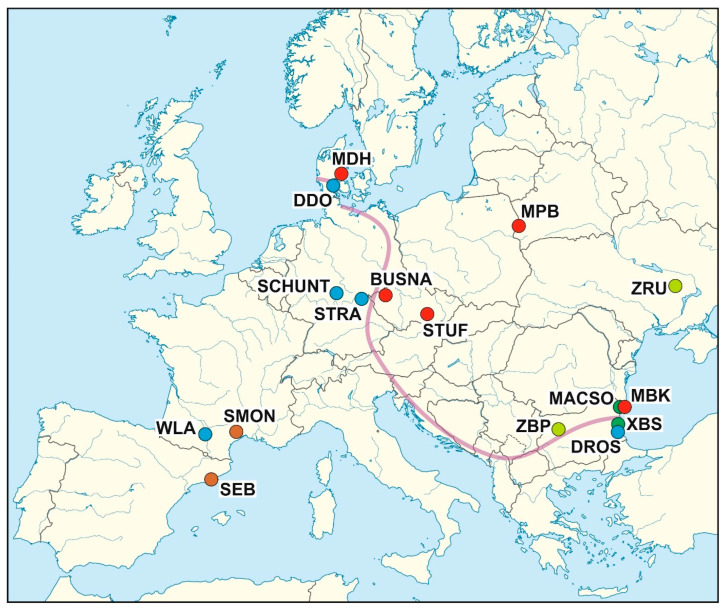
Map of Europe with locations of the founding populations of the WDSs employed in this study: brown circles: *M. spretus*, light green circles: *M. spicilegus*, dark green circles: *M. macedonicus*, red circles: *M. musculus musculus*, blue circles: *M. m. domesticus*; the violet line schematically depicts the hybrid zone between *M. m. musculus* and *M. m. domesticus*. Two *M. m. castaneus* stocks, CIM from Masinagudi, India, and CKN from Nairobi, Kenya, are missing.

Genomic DNA was extracted from the liver as described in the Supplementary Material. In total, we analysed 52 individuals of 18 WDSs, representing *M. m. musculus* (5 WDSs), *M. m. domesticus* (5 WDSs), *M. m. castaneus*, *M. spretus*, *M. macedonicus*, and *M. spicilegus* (2 WDSs each) (Table 1, Figure 1; also see Supplementary Material).

### 2.2. CNV

Copy numbers were scored using the QX200 Droplet Digital PCR System (Bio-Rad, Hercules, CA, USA). We designed an MUP-specific assay consisting of two primers and a fluorescent probe (Supplementary Figure S1, Supplementary Material) using C57BL/6J sequence and primer design tools (Geneious Prime 9.1.5, Biomatters, Auckland, New Zealand). The validity of the assay was checked first using NCBI Primer-BLAST (Supplementary Table S1) and then using C57BL/6J genomic DNA as a template. All samples were run in triplicates (technical replicates) and processed in the Quantasoft environment provided with the QX200 ddPCR System, and the resulting values were rounded. The resulting numbers were halved to obtain haploid CN estimates. Values for the biological replicates were then averaged.

### 2.3. Proteomic Analysis

We followed a protocol described in [71]. In short, all urine samples were precipitated with ice-cold acetone and centrifuged at 14,000 rpm for 10 min at 0 °C. The protein concentration of each lysate was determined using a BCA assay kit (Fisher Scientific, North Shore City, New Zealand). Peptides cleaved with trypsin were desalted on a Michrom C18 column. Nano reversed-phase columns were used (EASY-Spray column, 50 cm × 75 µm ID, PepMap C18, 2 µm particles, 100 Å pore size). Eluting peptide cations were converted to gas-phase ions via electrospray ionisation and analysed using Thermo Orbitrap Fusion (Q-OT-qIT, Thermo, Waltham, MA, USA) with the same parameters as described in [71,72,73].

LC-MS data were pre-processed with MaxQuant software (version 1.6.34) [74]. The false discovery rate (FDR) was set to 1% for both proteins and peptides, and we specified a minimum peptide length of seven amino acids. The Andromeda search engine was used for the MS/MS spectra mapping against the modified Uniprot *M. musculus* database, containing 44,900 entries. In this database, all original MUP sequences were replaced with a complete list of MUPs from the Ensembl database [75]. Quantifications were performed using label-free algorithms [74] with a combination of unique and razor peptides. From the whole-proteome dataset, we dissected concentrations (in ng/mL) of individual urinary MUPs, which were subsequently pooled.

### 2.4. Statistics

All datasets were tested for normal distribution. When no significant deviation from normality was proven, parametric tests (analysis of variance, Tukey HSD, Student’s *t*-test, Pearson’s correlation) were used; otherwise, non-parametric tests (Kruskal–Wallis, Mann–Whitney, median test) were applied. Statistica v. 14 [76] was employed for all the statistical analyses.

## 3. Results

### 3.1. CNV

**Figure 2:**
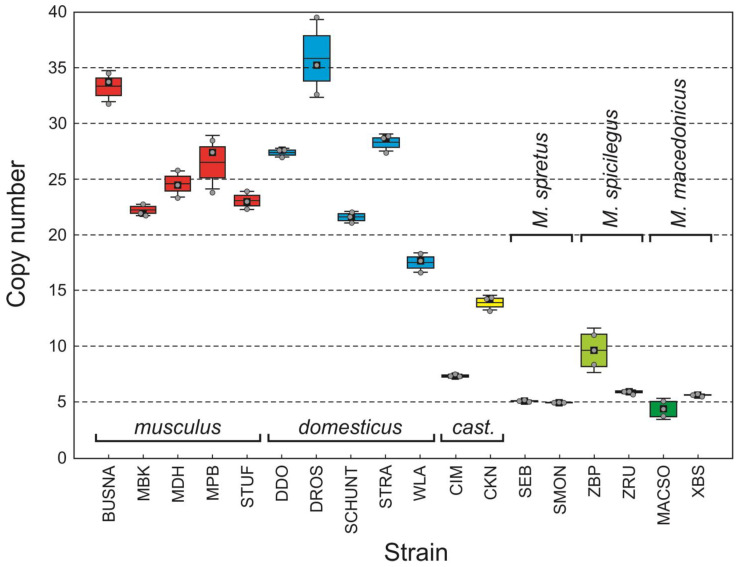
Box plot of copy numbers estimated for each WDS; horizontal line: mean; box: standard error; whiskers: standard deviation; black squares: median; *cast*.: *M. m. castaneus*; grey points depict raw data.

Variation within the technical (within-individual) replicates, expressed as Poisson errors provided by QX200 software, was very low (Supplementary Table S2), regardless of the number of copies (*r* = −0.102; *p* = 0.4916). Nevertheless, the variance between individuals within WDSs significantly increased with CN (Figure 2), i.e., the higher the CN, the greater the difference between individuals of the same strain (*r* = 0.541; *p* = 0.0204).

ANOVA revealed highly significant CN variation among the taxa (*F* = 44.649, d.f. = 5, *p* = 0.0000). As expected, non-commensal WDSs had the lowest CN captured by our assay, with average values ranging from 4.40 (MACSO) to 9.65 (ZPB), but mostly between 5 and 6 (Supplementary Table S2). In contrast, higher CN values were found in the commensal subspecies: the ranges of *Mup* copies were 22–35 in *M. m. musculus* (mean = 25.94, SE = 1.354), 17–40 in *M. m. domesticus* (mean = 26.11; SE = 2.093), and 7–14 in *M. m. castaneus* (mean = 10.62, SE = 1.486). According to the Tukey HSD post hoc test, the difference between *musculus* and *domesticus* was not significant (*p* = 1.0000), while both of the subspecies had significantly higher CNs than all other WDSs, including *castaneus* (*p* = 0.0001). Although *castaneus* also had a generally higher CN than the non-commensal species, these differences were not significant (*p* = 0.2960–0.8542 depending on comparison). Finally, differences between the non-commensal species were not significant (*p* = 0.9535–1.0000 depending on comparison) (Table S4, Supplementary Material).

As shown in Figure 2, CN values varied widely among the strains (*F* = 194.660, d.f. = 17, *p* = 0.0000), although the variation could only be tested in two commensal *M. musculus* subspecies, *musculus* and *domesticus*, for which a sufficient number of WDSs was measured. We gauged it as differences in average CN between WDSs within subspecies vs. differences between WDSs of different subspecies. Student’s *t*-tests showed no significant differences in both *musculus* and *domesticus* (*p* = 0.5012 and 0.1403, respectively), and the two subspecies did not significantly differ from each other in this respect (*p* = 0.0919). In summary, intrasubspecific variation appeared to be similar in the two subspecies, and it was not different from intersubspecific variation in both of them.

### 3.2. Total Urinary MUP Levels

**Figure 3:**
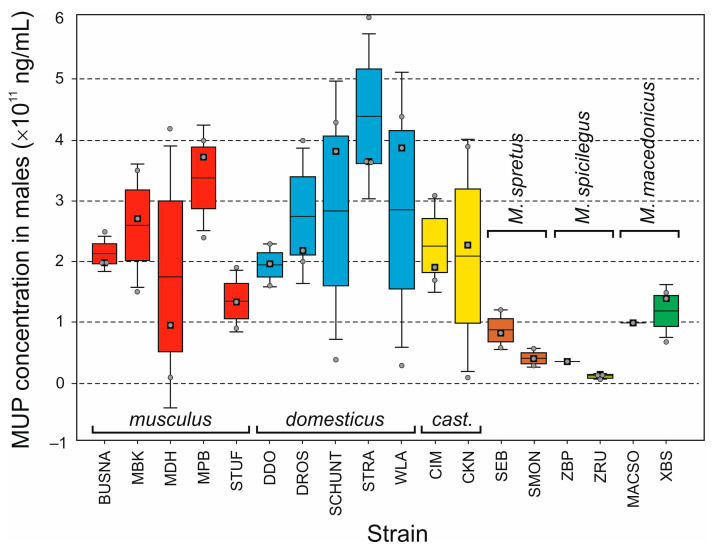
Box plot of total urinary MUP levels estimated for males of each WDS; horizontal line: mean; box: standard error; whiskers: standard deviation; black squares: median; *cast*.: *M. m. castaneus*; grey points depict raw data.

**Figure 4:**
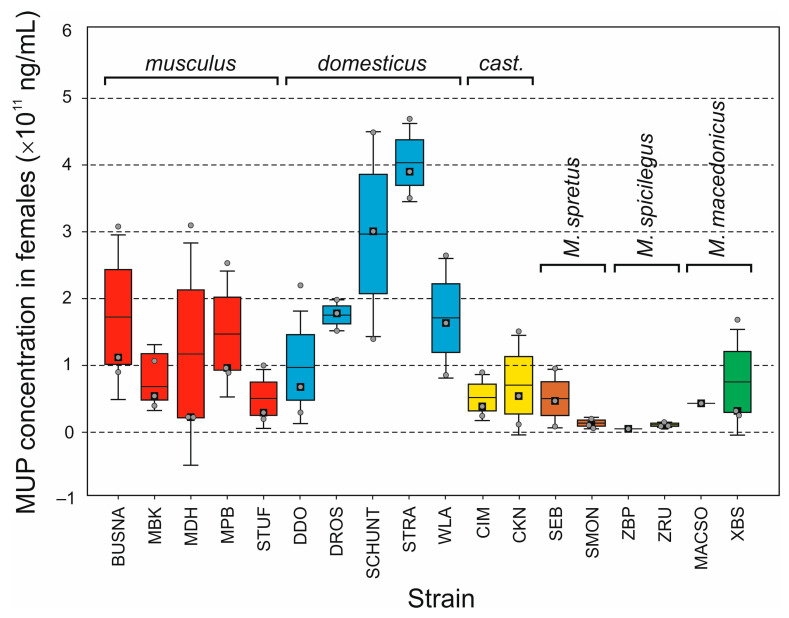
Box plot of total urinary MUP levels estimated for females of each WDS; horizontal line: mean; box: standard error; whiskers: standard deviation; black squares: median; cast.: M. m. castaneus; grey points depict raw data.

Like in CNV, the total uMUP levels varied considerably among WDSs, but, contrary to CNV, also within the strains, especially in *M. m. musculus* and *M. m. domesticus*, in both sexes (Figure 3 and Figure 4; Supplementary Table S6). Kruskal–Wallis and median tests of variance among strains yielded slightly different results, yet all were close to the significance limits (males: Kruskal–Wallis H(17,50) = 26.973, *p* = 0.0585; median test chi-square = 31.333, d.f. = 17, *p* = 0.0182; females: Kruskal–Wallis H(17,50) = 32.548, *p* = 0.0129; median test chi-square = 26.000, d.f. = 17, *p* = 0.0745). When taxa, instead of WDSs, were compared, all results were significant (males: Kruskal–Wallis H(5,50) = 21.135, *p* = 0.0008; median test chi-square = 20.667, d.f. = 5, *p* = 0.0009; females: Kruskal–Wallis H(5,50) = 25.0177, *p* = 0.0001; median test chi-square = 14,333, d.f. = 5, *p* = 0.0136). Kruskal–Wallis pairwise comparisons only showed significant differences between *musculus* and *M. spicilegus* in males (*p* = 0.0458) and between *domesticus*, *M. spretus*, and *M. spicilegus* in both sexes (males: *p* = 0.0213 and 0.0039, respectively; females: 0.0045 and 0.0008, respectively; Table S5, Supplementary Material). The same results were revealed using pairwise Mann–Whitney tests after Bonferroni adjustment of the α value.

Variation was again assessed only in *M. m. musculus* and *M. m. domesticus* as differences between WDSs within and between the subspecies. Significant differences between intrasubspecific and intersubspecific variation were found in neither subspecies nor sex (*p* > 0.05 in all cases). This means that mean differences between the consubspecific strains within *musculus* and *domesticus* are comparable to those between heterosubspecific strains. When we compared intrasubspecific variation between the subspecies, we found a non-significant result for males (*t*-test: *p* = 0.9556) but a significant result for females, with higher variation in *domesticus* than *musculus* (*p* = 0.0154).

To what extent does variation in the total uMUP amount reflect variation in CN? When all strains are pooled, the protein levels appear to be significantly correlated with CN (males: *r* = 0.699, *p* = 0.0015; females: *r* = 0.625, *p* = 0.0056). However, these results can be false positives caused by underlying general differences between the taxa, as shown in a hypothetical example in Supplementary Figure S2. Indeed, when the two taxa with sufficiently high *N* were tested separately, all results were insignificant (*musculus*, males: *r* = 0.118, *p* = 0.8505; females: *r* = 0.859, *p* = 0.0621; *domesticus*, males: *r* = 0.037, *p* = 0.9523; females: *r* = 0.046, *p* = 0.9420). In summary, uMUP CN is a poor predictor of the final protein excretion at the level of WDSs within the two subspecies.

## 4. Discussion

In this study, we have demonstrated that although the average copy number in *M. musculus* is generally higher than in its non-commensal relatives, CNV does not fully reflect the strength of synanthropy. This is evident as the CN of *M. m. castaneus*, on average, does not significantly differ from non-commensal species *M. spretus*, *M. macedonicus*, and *M. spicilegus*. Moreover, CN was a poor predictor of total uMUP excretion at the level of individual WDSs. Finally, we showed that variation among strains derived from different subspecies does not exceed variation within subspecies.

A low CN in *M. macedonicus*, *M. spicilegus*, and *M. spretus* is consistent with previously published results [52], suggesting that these non-commensal members of the *M. musculus* species group have only undergone one round of expansion of the central *Mup* gene cluster, while an additional expansion occurred in the three commensal house mouse subspecies prior to their diversification [52]. A higher CN can potentially allow finer diversification of chemical signals [77]. As pointed out by Hurst (1987) [53], Pocock et al. (2004) [78], and others, an ability to convey detailed individuality information through urine or other excreted fluids should be beneficial for enhanced interactions among animals in dense house mouse populations. Higher information complexity may thus have facilitated the evolution of human commensalism [52]. Similar associations between social structure and individuality levels have been observed in marmots [79]. It has also been shown that people from small home towns (i.e., from lower-density populations) have a poorer face-learning ability than individuals from large home towns (i.e., from higher-density populations) [80]. Moreover, a large part of this variation is genetically determined [81,82].

The strikingly low number of *Mup* genes found in the southeastern Asian house mouse subspecies *M. m. castaneus* is surprising since this taxon is commonly considered to be strongly synanthropic [61,65,67,68,69,70], with feral populations being extremely rare (e.g., in Micronesia [63]). It should also be noted that all of the *M. musculus*-derived strains used in this study, including the two *castaneus* WDSs (CIM, CKN), were established from commensal populations living in similar synanthropic habitats. Our study thus shows that the *Mup* CN is not correlated with the level of commensalism within the house mouse. This conclusion is reinforced by the considerable CN variance within each of the subspecies, indicating relaxed or weak selective pressure for a high CN.

The discrepancy between CN and final protein excretion is not unexpected for several reasons. First, in addition to functional *Mup* paralogs, CN estimated through ddPCR is likely to also involve several pseudogenes, despite our effort to only amplify functional genes. Second, the expression of individual MUPs is regulated by a complex endocrine system involving testosterone, thyroxine, and growth hormone [83,84]. Consequently, it can be down-regulated or up-regulated depending on health, food supply, age, and other conditions [85,86], or as a consequence of social interactions [87], although the latter potential influence might have been prevented or at least reduced by individual housing of the mice under study. It has been suggested that uMUPs are able to provide individual identity information in urine markings (e.g., [36,50,53,88,89,90,91,92]). This notion has recently been challenged by Thoß et al. [85,93], who argue that individual MUP excretion profiles are dynamic rather than stable over time, as required for the ‘barcode hypothesis’, implying a further cause of MUP excretion variability. In any case, regardless of which of the two contradictory hypotheses is correct, we should expect higher variation within WDSs in the total MUP levels than in the total *Mup* CN, in agreement with the results of this study.

At the level of the individual taxa, the overall uMUP excretion corresponds to the CN. The data obtained from the three non-commensal species are consistent with those published earlier [52,84], not only in terms of the apparently, though not always significantly, lower uMUP levels relative to *M. m. musculus* and *M. m. domesticus*, but also in terms of differences within the non-commensal group, with uMUP excretion being the highest in *M. macedonicus* and the lowest in *M. spicilegus* in both sexes (Figure 3 and Figure 4). In contrast, while previous studies [48,52,94] reported higher MUP levels in *M. m. musculus* males than in *M. m. domesticus* males, we found no significant differences between them. The cause of this discrepancy is unclear; however, it would be intriguing to investigate how different mouse subspecies respond to varying social contexts, which in this case was complete social isolation. On the other hand, our data are consistent with the lack of difference between these subspecies in females reported by Stopková et al. (2007) [48], Hurst et al. (2017) [94], and Sheehan et al. (2019) [52]. In fact, despite high variance among the consubspecific strains, we revealed slightly higher uMUP excretion in *domesticus* females than in *musculus* females, in agreement with Stopková et al. (2007) [48] and Sheehan et al. (2019) [52].

## 5. Conclusions

We have confirmed that WDSs harbour substantial genetic variability, making them highly valuable for many fields of life sciences, including copy number variation and its medicinal and evolutionary consequences. Chemical communication plays a crucial role in many mammals [95,96]. Consequently, some mammal species, such as the house mouse, have undergone extraordinary expansion of genes related to olfactory signals and their receptors [30,31,32]. Mouse olfactory signals involve products of various multigene families [33,34,35,97], which are released into various body fluids, mainly into urine. As a crucial part of urine scent marks [36,98], major urinary proteins have long been focused on in a vast array of studies (e.g., [36,52,84,90]). However, many of them employed highly inbred laboratory strains that had been created via artificial selection, and thus they substantially differed from wild mice in numerous traits [84]. Other surveys have been limited to a single subspecies. This all makes generalisations of such studies difficult.

Wild-derived strains offer the combined benefits of CLSs (reproducibility due to being inbred) and wild animals (natural variability). Here, we have demonstrated tremendous diversity in MUP gene copy number and total protein levels in urine samples obtained from mouse WDSs. This finding highlights the immense value of these stocks for genetic, biomedical, and evolutionary studies [17]. However, we should keep in mind that ‘riding two horses at once’, i.e., combining reproducibility with diversity, also imposes higher demands on the number and proper choice of employed WDSs. Namely, they should both compensate for the haphazardness of fixation of individual traits by the inbreeding process and cover as much of the natural variation as possible. The first demand can be met by choosing more than one WDS from the same local area, whereas the second calls for using stocks derived from populations scattered across a large portion of a (sub)species range.

To summarise, our investigation of *Mup* gene numbers and the levels of their final protein product in urine, using 18 stocks derived from commensal and non-commensal taxa of the *M. musculus* species group, suggests that the second expansion of the central *Mup* cluster is unlikely to facilitate a commensal relationship with humans in the three house mouse subspecies.

## Figures and Tables

**Table 1:** List of wild-derived strains used in this study. More details on the WDSs can be found at https://housemice.cz/en (accessed on 1 January 2021). *N* = number of individuals per strain (CNV/expression); note that CNV was estimated regardless of sex, whereas an equal number of males and females per strain was used for expression measurements.

		*M. musculus*						
*musculus*		*domesticus*			*castaneus*			
Strain	Country	*N*	Strain	Country	*N*	Strain	Country	*N*
BUSNA	Czechia	3/6	DDO	Denmark	3/6	CIM	India	3/6
MBK	Bulgaria	3/6	DROS	Bulgaria	3/6	CKN	Kenya	3/6
MDH	Denmark	3/6	SCHUNT	Germany	3/6			
MPB	Poland	3/6	STRA	Germany	3/6			
STUF	Czechia	3/6	WLA	France	3/6			
*M. spretus*		*M. spicilegus*			*M. macedonicus*			
Strain	Country	*N*	Strain	Country	*N*	Strain	Country	*N*
SEB	Spain	3/6	ZRU	Ukraine	3/6	XBS	Bulgaria	3/6
SMON	France	3/6	ZPB	Bulgaria	2/2	MACSO	Bulgaria	2/2

## Data Availability

All data analysed in this work are available in the Supplementary Material.

## Supplementary Materials

The following supporting information can be downloaded at https://www.mdpi.com/article/10.3390/genes14112090/s1: Figure S1: Position of ddPCR primers and probes in *Mup* paralogs (A) and the *Tert* gene (B). RP = reverse primer, FP = forward primer, E = exon. Proportions of exons, introns, primers, and probes are not exactly to scale; Figure S2: A hypothetical example of the discrepancy between correlations at different levels. While there is no correlation between the number of genes and their expression in Taxon 1, and this correlation is even significantly negative in Taxon 2, we detect a highly positive correlation in the total sample; Table S1: List of Mup paralogs covered by the Mup assay, with sequences of the primers and probes. The Mup paralogs are named according to the NCBI nomenclature. SNPs are marked in red; Table S2: List of Mup pseudogenes of lower specificity to the GRCm39, with sequences of the primers and probes. The loci are named according to the NCBI nomenclature; ‘Ps’ stands for pseudogene. SNPs are marked in red; Table S3: CN estimates for each individual studied; Table S4: The results of Tukey HSD post-hoc pairwise tests of estimated Mup CN between the mouse (sub)species: The *p*-values significant at the α = 0.05 level are marked in red; Table S5: The results of Kruskal-Wallis tests of the total MUP levels between the mouse (sub)species: *p*-values of multiple comparisons for males (top) and females (bottom). The values significant at the α = 0.05 level are marked in red; Table S6: Total MUP levels in the urine (ng/mL).
