# Supplementary material for: The Expansion of House Mouse Major Urinary Protein Genes Likely Did Not Facilitate Commensalism with Humans
Source: Genes (Basel). 2023 Nov 17;14(11):2090. doi: 10.3390/genes14112090 (PMC10671799; doi:10.3390/genes14112090)
Supplement: Supplementary file 1 [file genes-14-02090-s001.zip › genes-2689022-supplementary.pdf]

# Supplementary Material

## Mice

All mice were housed in a conventional breeding facility at the Institute of Vertebrate Biology, Czech Academy of Sciences, in Studenec. They were maintained under standard conditions, with a light:dark regime of 14:10 hours (light 6 am – 8 pm), temperature of 22–23 °C, and humidity within the range of 40-70%. Mice had access to standard food pellets (Myška 1, VKS Podhledští Dvořáci, Hamry, Czechia) and tap water *ad libitum*. Mice were weaned at 20 days of age and then housed individually in transparent polycarbonate cages equipped with sifted sawdust, shredded paper for nest building and nest houses made of red transparent polycarbonate. The cages were cleaned every 5-7 days. The two subspecies were kept in different rooms. This facility is authorised for the use of experimental animals (Licence MZE-50144/2022-13143), as well as for the breeding and supply of experimental animals to third parties (MZE-50151/2022-13143). These licences are in compliance with the corresponding regulations and standards of the European Union, as specified in Council Directive 86/609/EEC. All animals were handled by authorised persons only (Licence No. CZ01271 for Z.H.).

At the age ~100 days (range 60–140 days, Table S3; basic statistics of the age structure are available upon request from the corresponding author), the mice were sacrificed by cervical dislocation and immediately dissected. Parts of the liver were snap-frozen in liquid nitrogen and then stored at -80 °C until DNA extraction.

## Genomic DNA

About 3-mm$^3$ pieces of tissue were put in the 2-ml Reinforced Tubes with 2.8 mm Ceramic Beads (Omni International), 360 µl of the T1 Lysis Buffer (Macherey-Nagel) and 50 µl of Proteinase-K (Qiagen), homogenised using the MagNA Lyser Instrument (Roche), and incubated for 3–6 h at 56 °C in a shaker (200 rpm). Genomic DNA was then isolated using the NucleoSpin kit (Macherey-Nagel) following the manufacturer's instructions, with the final ddH$_2$O elution. The concentration and purity of the extraction products were checked with a spectrophotometer (DeNovix DS 11). Subsequently, all samples were equalised to contain 50–100 copies of the reference gene per µl in each ddPCR well (see below).

## *Mup* Assay

The *Mup* assay was designed based on ~2.5 Mb long C57BL/6 sequence of Chromosome 4 (NC_000070.7) bracketing the *Mup* cluster downloaded from NCBI GRCm38.p4 genome assembly. Geneious 9.1.5 (Biomatters) was used for the identification of *Mup* paralogs and pseudogenes as well as for designing primers and probes. The assay focused on the central *Mup* genes, on areas with the lowest SNP density and highest difference from pseudogenes. As the primer-design tool implemented in Geneious rendered several potential assays, we tested the proposed oligonucleotides with the online tool Oligo-Analyzer (Integrated DNA Technologies, IA, USA), retaining the two best candidates (pair of primers + probe). Finally, we used NCBI's Primer-BLAST tool (http://www.ncbi.nlm.nih.gov/tools/primer-blast) to check the specificity (Tables S1 and S2) using the GRCm39 built. The resulting assay 'Mup_E5_S1' (Exone 5, set 1; '*Mup* assay' in short) (Fig. S1A) was purchased as a custom-designed PrimeTime qPCR Assay (IDT, IA, USA) in primer-to-probe ratio 3.6 and resuspended upon delivery 20× with the recommended buffer.

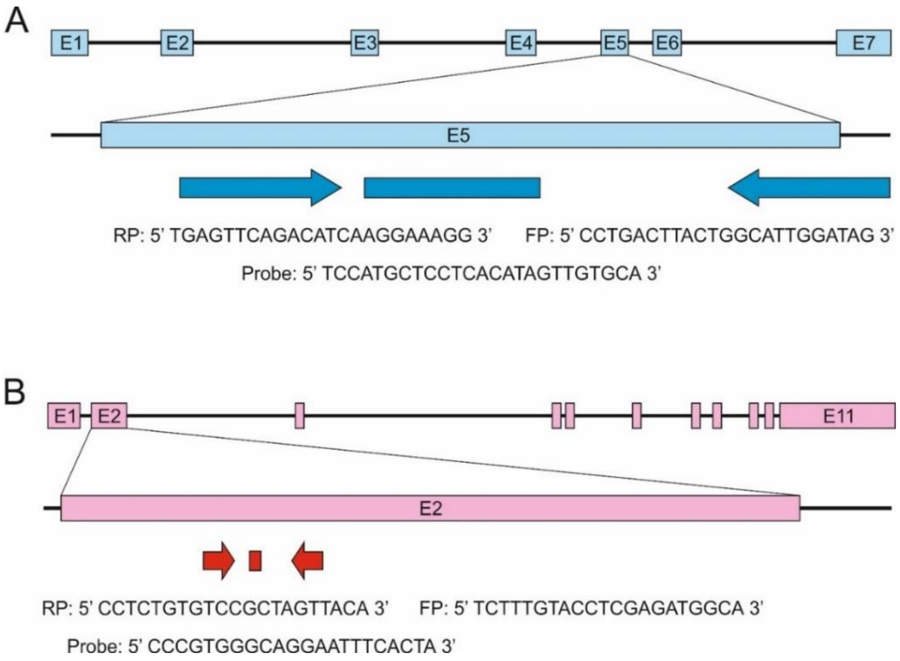


**Fig. S1 Position of ddPCR primers and probes in *Mup* paralogs (A) and the *Tert* gene (B).** RP = reverse primer, FP = forward primer, E = exon. Proportions of exons, introns, primers, and probes are not exactly to scale.

## ddPCR

The telomerase reverse transcriptase gene (*Tert*), which is known to be present in two copies per diploid genome (Pezer et al. 2015), was used as standard (primers and probe shown in Fig. S1B). The ddPCR reactions were run in duplexes involving both the target (*Mup*, FAM-labelled) and standard (*Tert*, HEX-labelled). The *Mse*I restriction endonuclease was used to ensure an independent separation of *Mup* paralogs. The reaction mixture comprised 12 µl of ddPCR Supermix for Probes (Bio-Rad), 0.12 µl of the *Mup* assay, 0.12 µl of the *Tert* assay, 0.05 µl of *Mse*I 10 000 U/ml (New England Biolabs, Ipswich, MA, USA), 1.1 µl of genomic DNA (in some cases, the volume was adjusted to produce a desired load 50–100 copies/µl in the well), and $ddH_2O$ to the final volume 24 µl.

Before emulsion, the mixture was incubated at 37 °C for 10 min as recommended by the manufacturer (Bio-Rad 2015) ) and verified in an earlier study (Yanchukov et al., 2021, https://doi.org/10.1101/2021.12.22.473661). After the incubation, emulsions of droplets were prepared with the QX200 Droplet Generator (Bio-Rad). PCR reaction was then carried out in the Bio-Rad C1000 cycler using a deep-well block. Droplet fluorescence was screened in the QX200 Droplet Reader (Bio-Rad). Initially, several thermal gradients were tried to find the temperature at which both the target and standard showed sufficiently clear positive vs. negative droplet separation, needed for reliable CN estimation. The definitive conditions were: 10 min at 95 °C, followed by 40 two-step cycles with 30 sec at 94 °C and 1 min at 59 °C, with the final extension of 10 min at 98 °C. The ramp speed was set to 2 °C/sec for all steps and cycles.

**Table S1. List of Mup paralogs covered by the Mup assay, with sequences of the primers and probes.**
The Mup paralogs are named according to the NCBI nomenclature. SNPs are marked in red.

| Paralog | Primers, 5´ → 3´ | Probe, 5´ → 3´ |
|---|---|---|
| *Mup2* | CCTGACTTACTGGCATTGGATAG | TCCATGCTCCTCACATAGTTGTGCA |
| #1 | TGAGTTCAGACATCAAGGAAAGG | |
| *Mup8* | CCTGACTTACTGGCATTGGATAG | TCCATGCTCCTCACATAGTTGTGCA |
| #2 | TGAGTTCAGACATCAAGGAAAGG | |
| *Mup9* | CCTGACTTACTGGCATTGGATAG | TCCATGCTCCTCACATAGTTGTGCA |
| #3 | TGAGTTCAGACATCAAGGAAAGG | |
| *Mup10* | CCTGACTTACTGGCATTGGATAG | TCCATGCTCCTCACATAGTTGTGCA |
| #4 | TGAGTTCAGACATCAAGGAAAGG | |
| *Mup11* | CCTGACTTACTGGCATTGGATAG | TCCATGCTCCTCACATAGTTGTGCA |
| #5 | TGAGTTCAGACATCAAGGAAAGG | |
| *Mup22* | CCTGACTTACTGGCATTGGATAG | TCCATGCTCCTCACATAGTTGTGCA |
| #6 | TGAGTTCAGACATCAAGGAAAGG | |
| *Mup13* | CCTGACTTACTGGCATTGGATAG | TCCATGCTCCTCACATAGTTGTGCA |
| #7 | TGAGTTCAGACATCAAGGAAAGG | |
| *Mup14* | CCTGACTTACTGGCATTGGATAG | TCCATGCTCCTCACATAGTTGTGCA |
| #8 | TGAGTTCAGACATCAAGGAAAGG | |
| *Mup16* | CCTGACTTACTGGCATTGGATAG | TCCATGCTCCTCACATAGTTGTGCA |
| #9 | TGAGTTCAGACATCAAGGAAAGG | |
| *Mup17* | CCTGACTTACTGGCATTGGATAG | TCCATGCTCCTCACATAGTTGTGCA |
| #10 | TGAGTTCAGACATCAAGGAAAGG | |
| *Mup18* | CCTGACTTACTGGCATTGGATAG | TCCATGCTCCTCACATAGTTGTGCA |
| #11 | TGAGTTCAGACATCAAGGAAAGG | |
| *Mup19* | CCTGACTTACTGGCATTGGATAG | TCCATGCTCCTCACATAGTTGTGCA |
| #12 | TGAGTTCAGACATCAAGGAAAGG | |
| *Mup7* | CCTGACTTACTGGCATTGGATAG | TCCATGCT**T**CTCACATAGTTGTGCA |
| #13 | TGAGTTCAGACATCAAGGAAAGG | |
| *Mup1* | CCTGACTTACTGGCATTGGATAG | TCCATGCT**T**CTCACATAGTTGTGCA |
| #14 | TGAGTTCAGACATCAAGGAAAGG | |
| *Mup12* | CCTGACTTACTGGCATTGGATAG | TCCATGCT**T**CTCACATAGTTGTGCA |
| #15 | TGAGTTCAGACATCAAGGAAAGG | |
| *Mup15* | CCTGACTTACTGGCATTGGATAG | TCCATGCT**T**CTCACATAGTTGTGCA |
| #16 | TGAGTTCAGACATCAAGGAAAGG | |
| *Mup5* | CCTGACTTACTGGCATTGGATAG | TCCATGCTCCTCACATAGTT**T**TGCA |
| #17 | TGAGTTCAGACATCAAGGAAA**A**G | |
| *MupPs19* | CCTGACTTACTGGCATTGG**T**TAG | TCCATGCTCCTC**G**C**T**TAGTTGTGCA |
| #18 | TGAGTTCAGACATCAAGGAAA**A**G | |
| *MupPs21* | CCTGACTTACTGGCATTGG**T**TAG | TCCATGCTCCTC**G**C**T**TAGTTGTGCA |
| #19 | TGAGTTCAGACATCAAGGAAA**A**G | |
| *Mup20* | CCTGACTTACTGGCATTGG**T**TAG | TCCATGCTCCTC**G**C**T**TAGTTGTGCA |
| #20 | TGAGTTCAGACATCAAGGAAA**A**G | |

**Table S2. List of Mup pseudogenes of lower specificity to the GRCm39, with sequences of the primers and probes.** The loci are named according to the NCBI nomenclature; 'Ps' stands for pseudogene. SNPs are marked in red.

| Paralog | Primers, 5´ → 3´ | Probe, 5´ → 3´ |
|---|---|---|
| *MupPs18* | CCTGAGTTACTGGCATTGGATAG | TGCATGCTCCTCACATAATTTTGCA |
| | TGAGTTCAGACATCAAGGAAAAG | |
| *MupPs12* | CCTGACTTACTGGCATTAGATAG | TCCATGCTCCTCACATAGTTTTTCA |
| | TGAGTTCAGACATCAAGGAAGAG | |
| *MupPs1* | CCTGACTTACTGGCATTAGATAG | TCCATGCTCCTCAAATAGTTTTGCA |
| | TGAGTTCAGACATCAAGGAAGAG | |
| *MupPs2* | CCTGACTTACTGGCATTAGATAG | TCCATGCTCCTCAAATAGTTTTGCA |
| | TGAGTTCAGACATCAAGGAAGAG | |
| *MupPs10* | CCTGACTTACTGGCATTAGATAG | TCCATGCTCCTCAAATAGTTTTGCA |
| *LOC108168721* | TGAGTTCAGACATCAAGGAAGAG | |

## CNV estimation

All samples were run in triplicates (technical replicates), and data obtained were processed in the Quantasoft Software (Bio-Rad). For optimisation, we employed the same laboratory strain as we used for designing the *Mup* assay, i.e. C57BL/6.

On each plate, we used C57BL/6 gDNA as a positive control and for setting the threshold. The Quantasoft Software was employed to merge the triplicates, estimate CN, and calculate Poisson measurement errors (see Table S3). Only estimates with errors of less than 10% were accepted as valid.

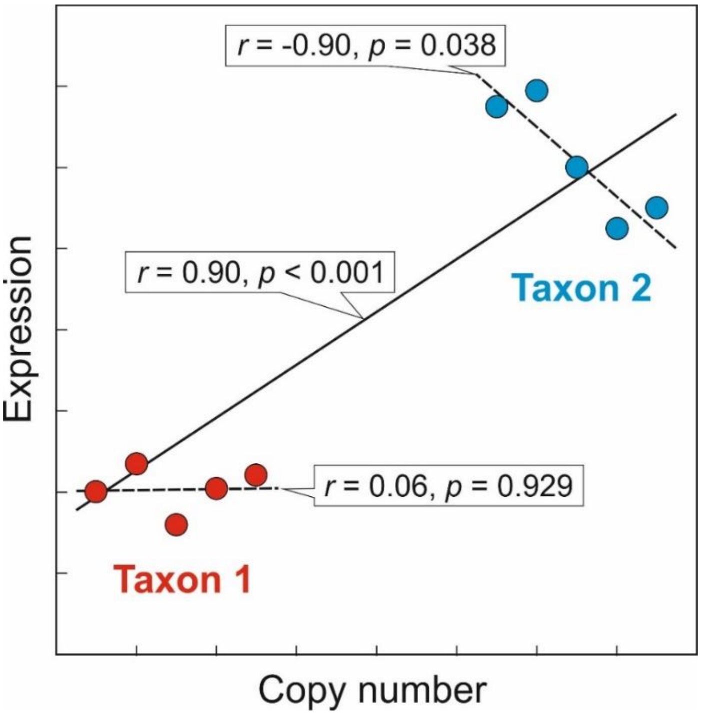


**Fig. S2 A hypothetical example of the discrepancy between correlations at different levels**. While there is no correlation between the number of genes and their expression in Taxon 1, and this correlation is even significantly *negative* in Taxon 2, we detect a highly *positive* correlation in the total sample.

**Table S3. CN estimates for each individual studied.**

| ID | Taxon | WDS | Age (days) | Total CN | +Poisson error | -Poisson error |
|---|---|---|---|---|---|---|
| SZ♂0922 | *M. m. musculus* | BUSNA | 140 | 34 | 1.3 | 1.3 |
| SZ♂8455 | *M. m. musculus* | BUSNA | 98 | 32 | 1.5 | 1.5 |
| SZ♂8456 | *M. m. musculus* | BUSNA | 98 | 35 | 1.8 | 1.7 |
| SZ♂7552 | *M. m. musculus* | MBK | 80 | 23 | 0.9 | 0.9 |
| SZ♂8569 | *M. m. musculus* | MBK | 119 | 22 | 0.9 | 0.9 |
| SZ♀1605 | *M. m. musculus* | MBK | 136 | 22 | 0.9 | 0.8 |
| SZ♂1561 | *M. m. musculus* | MDH | 139 | 26 | 1.1 | 1.1 |
| SZ♂7092 | *M. m. musculus* | MDH | 99 | 23 | 1.1 | 1.1 |
| SZ♂7832 | *M. m. musculus* | MDH | 134 | 24 | 0.8 | 0.8 |
| SZ♂0788 | *M. m. musculus* | MPB | 62 | 24 | 1.0 | 0.9 |
| SZ♂7398 | *M. m. musculus* | MPB | 88 | 27 | 1.2 | 1.1 |
| SZ♂8772 | *M. m. musculus* | MPB | 111 | 28 | 1.3 | 1.2 |
| SZ♂2157 | *M. m. musculus* | STUF | 113 | 22 | 0.9 | 0.9 |
| SZ♂8789 | *M. m. musculus* | STUF | 84 | 24 | 1.2 | 1.2 |
| SZ♂8790 | *M. m. musculus* | STUF | 110 | 23 | 1.0 | 0.9 |
| SZ♂1155 | *M. m. domesticus* | DDO | 131 | 27 | 1.1 | 1.1 |
| SZ♂7605 | *M. m. domesticus* | DDO | 108 | 28 | 1.1 | 1.1 |
| SZ♂7612 | *M. m. domesticus* | DDO | 108 | 28 | 1.1 | 1.1 |
| SZ♂7305 | *M. m. domesticus* | DROS | 87 | 35 | 1.6 | 1.6 |
| SZ♂8265 | *M. m. domesticus* | DROS | 106 | 40 | 1.6 | 1.6 |
| SZ♀8268 | *M. m. domesticus* | DROS | 106 | 33 | 1.3 | 1.3 |
| SZ♂1005 | *M. m. domesticus* | SCHUNT | 136 | 22 | 0.8 | 0.7 |
| SZ♂7274 | *M. m. domesticus* | SCHUNT | 69 | 21 | 0.9 | 0.9 |
| SZ♂7405 | *M. m. domesticus* | SCHUNT | 60 | 22 | 0.9 | 0.8 |
| SZ♂1363 | *M. m. domesticus* | STRA | 124 | 29 | 1.1 | 1.1 |
| SZ♂7484 | *M. m. domesticus* | STRA | 84 | 29 | 1.4 | 1.3 |
| SZ♂7700 | *M. m. domesticus* | STRA | 102 | 27 | 1.1 | 1.0 |
| SZ♂1907 | *M. m. domesticus* | WLA | 122 | 18 | 0.8 | 0.7 |
| SZ♂7156 | *M. m. domesticus* | WLA | 96 | 17 | 0.6 | 0.6 |
| TA♂0051 | *M. m. domesticus* | WLA | 85 | 18 | 0.8 | 0.6 |
| SZ♂2484 | *M. m. castaneus* | CIM | 120 | 7 | 0.3 | 0.2 |
| SZ♂2486 | *M. m. castaneus* | CIM | 100 | 7 | 0.4 | 0.3 |
| SZ♂9032 | *M. m. castaneus* | CIM | 100 | 8 | 0.3 | 0.2 |
| SZ♂1551 | *M. m. castaneus* | CKN | 118 | 14 | 0.7 | 0.6 |
| SZ♂8515 | *M. m. castaneus* | CKN | 95 | 13 | 0.5 | 0.6 |
| SZ♀8517 | *M. m. castaneus* | CKN | 95 | 14 | 0.7 | 0.5 |
| SZ♂1527 | *M. spretus* | SEB | 118 | 5 | 0.2 | 0.2 |
| SZ♂7696 | *M. spretus* | SEB | 102 | 5 | 0.2 | 0.2 |
| SZ♂8111 | *M. spretus* | SEB | 115 | 5 | 0.2 | 0.2 |
| SZ♂0722 | *M. spretus* | SMON | 65 | 5 | 0.2 | 0.2 |
| SZ♂1620 | *M. spretus* | SMON | 136 | 5 | 0.2 | 0.2 |
| SZ♂7048 | *M. spretus* | SMON | 81 | 5 | 0.2 | 0.2 |
| SZ♂2268 | *M. spicilegus* | ZPB | 110 | 8 | 0.4 | 0.4 |
| SZ♀2271 | *M. spicilegus* | ZPB | 110 | 11 | 0.4 | 0.5 |
| SZ♂6098 | *M. spicilegus* | ZRU | 123 | 6 | 0.3 | 0.2 |
| SZ♂6187 | *M. spicilegus* | ZRU | 62 | 6 | 0.3 | 0.3 |
| SZ♀8325 | *M. spicilegus* | ZRU | 129 | 6 | 0.2 | 0.2 |

| | | | | | | |
|---|---|---|---|---|---|---|
| SZ♂1952 | *M. macedonicus* | MACSO | 121 | 5 | 0.3 | 0.3 |
| SZ♀3157 | *M. macedonicus* | MACSO | 91 | 4 | 0.2 | 0.1 |
| SZ♂0793 | *M. macedonicus* | XBS | 61 | 6 | 0.3 | 0.2 |
| TA♂0671 | *M. macedonicus* | XBS | 99 | 6 | 0.3 | 0.2 |
| TA♂1148 | *M. macedonicus* | XBS | 77 | 6 | 0.3 | 0.3 |

**Table S4. The results of Tukey HSD post-hoc pairwise tests of estimated Mup CN between the mouse (sub)species:** The p-values significant at the alpha = 0.05 level are marked in red.

| | *mus* | *dom* | *cas* | *spr* | *spi* | *mac* |
|---|---|---|---|---|---|---|
| *M. m. musculus* | -- | | | | | |
| *M. m. domesticus* | 1.0000 | -- | | | | |
| *M. m. castaneus* | 0.0001 | 0.0001 | -- | | | |
| *M. spretus* | 0.0001 | 0.0001 | 0.2960 | -- | | |
| *M. spicilegus* | 0.0001 | 0.0001 | 0.8542 | 0.9535 | -- | |
| *M. macedonicus* | 0.0001 | 0.0001 | 0.3675 | 1.0000 | 0.9679 | -- |

**Table S5. The results of Kruskal-Wallis tests of the total MUP levels between the mouse (sub)species:** p-values of multiple comparisons for males (top) and females (bottom). The values significant at the alpha = 0.05 level are marked in red.

| | *mus* | *dom* | *cas* | *spr* | *spi* | *mac* |
|---|---|---|---|---|---|---|
| *M. m. musculus* | -- | | | | | |
| *M. m. domesticus* | 1.0000 | -- | | | | |
| *M. m. castaneus* | 1.0000 | 1.0000 | -- | | | |
| *M. spretus* | 0.2556 | 0.0213 | 0.9828 | -- | | |
| *M. spicilegus* | 0.0458 | 0.0039 | 0.2177 | 1.0000 | -- | |
| *M. macedonicus* | 1.0000 | 0.6235 | 1.0000 | 1.0000 | 1.0000 | -- |

| | *mus* | *dom* | *cas* | *spr* | *spi* | *mac* |
|---|---|---|---|---|---|---|
| *M. m. musculus* | -- | | | | | |
| *M. m. domesticus* | 0.4538 | -- | | | | |
| *M. m. castaneus* | 1.0000 | 0.1403 | -- | | | |
| *M. spretus* | 0.7177 | 0.0045 | 1.0000 | -- | | |
| *M. spicilegus* | 0.1269 | 0.0008 | 1.0000 | 1.0000 | -- | |
| *M. macedonicus* | 1.0000 | 0.7926 | 1.0000 | 1.0000 | 1.0000 | -- |

**Table S6. Total MUP levels in the urine (ng/ml).**

| Taxon | WDS | Sex | Total MUP |
|---|---|---|---|
| *M. m. musculus* | BUSNA | male | 2.46517E+11 |
| *M. m. musculus* | BUSNA | male | 1.98267E+11 |
| *M. m. musculus* | BUSNA | male | 1.94918E+11 |
| *M. m. musculus* | BUSNA | female | 90922726300 |
| *M. m. musculus* | BUSNA | female | 1.11731E+11 |
| *M. m. musculus* | BUSNA | female | 3.14495E+11 |
| *M. m. musculus* | MBK | male | 3.55171E+11 |
| *M. m. musculus* | MBK | male | 2.71233E+11 |
| *M. m. musculus* | MBK | male | 1.54604E+11 |
| *M. m. musculus* | MBK | female | 1.07276E+11 |
| *M. m. musculus* | MBK | female | 41659054400 |
| *M. m. musculus* | MBK | female | 54580663570 |
| *M. m. musculus* | MDH | male | 4.18523E+11 |
| *M. m. musculus* | MDH | male | 11293057560 |
| *M. m. musculus* | MDH | male | 96256335100 |
| *M. m. musculus* | MDH | female | 3.09352E+11 |
| *M. m. musculus* | MDH | female | 21681455230 |
| *M. m. musculus* | MDH | female | 20432230660 |
| *M. m. musculus* | MPB | male | 2.39306E+11 |
| *M. m. musculus* | MPB | male | 3.7173E+11 |
| *M. m. musculus* | MPB | male | 4.02615E+11 |
| *M. m. musculus* | MPB | female | 88221641470 |
| *M. m. musculus* | MPB | female | 2.55394E+11 |
| *M. m. musculus* | MPB | female | 96296607800 |
| *M. m. musculus* | PWD | male | 2.58023E+11 |
| *M. m. musculus* | PWD | male | 3.01607E+11 |
| *M. m. musculus* | PWD | female | 29577503510 |
| *M. m. musculus* | PWD | female | 72959030670 |
| *M. m. musculus* | STUF | male | 1.86953E+11 |
| *M. m. musculus* | STUF | male | 1.33419E+11 |
| *M. m. musculus* | STUF | male | 85705173340 |
| *M. m. musculus* | STUF | female | 1.00333E+11 |
| *M. m. musculus* | STUF | female | 28554232670 |
| *M. m. musculus* | STUF | female | 21152495230 |
| *M. m. domesticus* | DDO | male | 1.58505E+11 |
| *M. m. domesticus* | DDO | male | 1.96881E+11 |
| *M. m. domesticus* | DDO | male | 2.28704E+11 |
| *M. m. domesticus* | DDO | female | 31608436030 |
| *M. m. domesticus* | DDO | female | 67062170000 |
| *M. m. domesticus* | DDO | female | 1.92464E+11 |
| *M. m. domesticus* | DROS | male | 2.03232E+11 |
| *M. m. domesticus* | DROS | male | 4.03498E+11 |
| *M. m. domesticus* | DROS | male | 2.18228E+11 |
| *M. m. domesticus* | DROS | female | 1.51353E+11 |
| *M. m. domesticus* | DROS | female | 1.77458E+11 |
| *M. m. domesticus* | DROS | female | 1.97522E+11 |
| *M. m. domesticus* | SCHUNT | male | 4.31599E+11 |

| | | | |
|---|---|---|---|
| *M. m. domesticus* | SCHUNT | male | 39843145360 |
| *M. m. domesticus* | SCHUNT | male | 3.81514E+11 |
| *M. m. domesticus* | SCHUNT | female | 1.40508E+11 |
| *M. m. domesticus* | SCHUNT | female | 4.47635E+11 |
| *M. m. domesticus* | SCHUNT | female | 3.01116E+11 |
| *M. m. domesticus* | STRA | male | 5.94708E+11 |
| *M. m. domesticus* | STRA | male | 3.64625E+11 |
| *M. m. domesticus* | STRA | male | 3.57001E+11 |
| *M. m. domesticus* | STRA | female | 3.52241E+11 |
| *M. m. domesticus* | STRA | female | 4.67474E+11 |
| *M. m. domesticus* | STRA | female | 3.90032E+11 |
| *M. m. domesticus* | WLA | male | 3.8744E+11 |
| *M. m. domesticus* | WLA | male | 4.43308E+11 |
| *M. m. domesticus* | WLA | male | 25712726630 |
| *M. m. domesticus* | WLA | female | 84959764600 |
| *M. m. domesticus* | WLA | female | 1.63757E+11 |
| *M. m. domesticus* | WLA | female | 2.63347E+11 |
| *M. m. castaneus* | CIM | male | 1.72722E+11 |
| *M. m. castaneus* | CIM | male | 1.92545E+11 |
| *M. m. castaneus* | CIM | male | 3.13676E+11 |
| *M. m. castaneus* | CIM | female | 90957106500 |
| *M. m. castaneus* | CIM | female | 38601120090 |
| *M. m. castaneus* | CIM | female | 25533672800 |
| *M. m. castaneus* | CKN | male | 2.27304E+11 |
| *M. m. castaneus* | CKN | male | 11024921640 |
| *M. m. castaneus* | CKN | male | 3.91792E+11 |
| *M. m. castaneus* | CKN | female | 5617526230 |
| *M. m. castaneus* | CKN | female | 53824091000 |
| *M. m. castaneus* | CKN | female | 1.51756E+11 |
| *M. spretus* | SEB | male | 82471517000 |
| *M. spretus* | SEB | male | 58464708700 |
| *M. spretus* | SEB | male | 1.22234E+11 |
| *M. spretus* | SEB | female | 47492290000 |
| *M. spretus* | SEB | female | 94988258550 |
| *M. spretus* | SEB | female | 7644034850 |
| *M. spretus* | SMON | male | 41354940440 |
| *M. spretus* | SMON | male | 57883152100 |
| *M. spretus* | SMON | male | 26390689530 |
| *M. spretus* | SMON | female | 22936536000 |
| *M. spretus* | SMON | female | 10536046730 |
| *M. spretus* | SMON | female | 7388313180 |
| *M. spicilegus* | ZBP | male | 35700809700 |
| *M. spicilegus* | ZBP | female | 5217857200 |
| *M. spicilegus* | ZRU | male | 6674888600 |
| *M. spicilegus* | ZRU | male | 14893201300 |
| *M. spicilegus* | ZRU | male | 14137819000 |
| *M. spicilegus* | ZRU | female | 9291124830 |
| *M. spicilegus* | ZRU | female | 15389113500 |
| *M. spicilegus* | ZRU | female | 8772840360 |
| *M. macedonicus* | MACSO | male | 98631559600 |

| | | | |
|---|---|---|---|
| *M. macedonicus* | MACSO | female | 43432965500 |
| *M. macedonicus* | XBS | male | 1.48994E+11 |
| *M. macedonicus* | XBS | male | 68465748000 |
| *M. macedonicus* | XBS | male | 1.37363E+11 |
| *M. macedonicus* | XBS | female | 1.66277E+11 |
| *M. macedonicus* | XBS | female | 32202607900 |
| *M. macedonicus* | XBS | female | 26921836039 |
